# A Comprehensive Analysis on the Association between Tobacco-Free Betel Quid and Risk of Head and Neck Cancer in Taiwanese Men

**DOI:** 10.1371/journal.pone.0164937

**Published:** 2016-10-25

**Authors:** Yuan-Hua Wu, Chia-Jui Yen, Jenn-Ren Hsiao, Chun-Yen Ou, Jehn-Shyun Huang, Tung-Yiu Wong, Sen-Tien Tsai, Cheng-Chih Huang, Wei-Ting Lee, Ken-Chung Chen, Sheen-Yie Fang, Jiunn-Liang Wu, Wei-Ting Hsueh, Forn-Chia Lin, Ming-Wei Yang, Jang-Yang Chang, Hsiao-Chen Liao, Shang-Yin Wu, Chen-Lin Lin, Yi-Hui Wang, Ya-Ling Weng, Han-Chien Yang, Yu-Shan Chen, Jeffrey S. Chang

**Affiliations:** 1 Department of Radiation Oncology, National Cheng Kung University Hospital, College of Medicine, National Cheng Kung University, Tainan, Taiwan; 2 Division of Hematology/Oncology, Department of Internal Medicine, National Cheng Kung University Hospital, College of Medicine, National Cheng Kung University, Tainan, Taiwan; 3 Department of Otolaryngology, National Cheng Kung University Hospital, College of Medicine, National Cheng Kung University, Tainan, Taiwan; 4 Department of Stomatology, National Cheng Kung University Hospital, College of Medicine, National Cheng Kung University, Tainan, Taiwan; 5 National Institute of Cancer Research, National Health Research Institutes, Tainan, Taiwan; 6 Department of Nursing, National Cheng Kung University Hospital, College of Medicine, National Cheng Kung University, Tainan, Taiwan; Fu Jen Catholic University, TAIWAN

## Abstract

**Objectives:**

Although betel quid (BQ) is an established risk factor of head and neck cancer (HNC), insufficiencies exist in the literature regarding the dose-response, BQ types, HNC sites, and BQ cessation. The current study was conducted to fill these insufficiencies.

**Materials and Methods:**

A hospital-based case-control study was conducted to evaluate the association between BQ and HNC. In-person interview was conducted to collect data on BQ chewing. The current analysis included 487 men newly diagnosed with HNC and 617 male controls who were frequency-matched to the cases by age. The association between BQ and HNC was assessed using multivariable unconditional logistic regression.

**Results:**

Ever BQ chewing was associated with an increased HNC risk regardless of the BQ types. A non-linear positive association between BQ and HNC was observed, with a steep rise in HNC risk for the first 5 pack-years or 200,000 minutes of BQ consumption. Every year of BQ cessation was associated with a 2.9% reduction in HNC risk; however, the risk did not reduce to the level of non-BQ chewers even after 20 years of BQ cessation. Eliminating BQ chewing may prevent 51.6% of HNCs, 62.6% of oral cancers, and 41.3% of pharyngeal cancers in Taiwan.

**Conclusion:**

Our results supported the positive association between BQ and HNC. BQ cessation is effective in reducing HNC risk and should be encouraged. Because BQ cessation may not reduce the HNC risk to the level of non-BQ chewers, it is important to prevent the initiation of BQ chewing.

## Introduction

Head and neck cancer (HNC), including cancers of the oral cavity, oropharynx, hypopharynx, and larynx, is one of the leading cancers in the world, with approximately 600,000 new cases each year [1]. HNC was the fourth most common cancer among Taiwanese men in 2011 with the incidence rising from 5 per 100,000 in 1979 to 41 per 100,000 in 2011 [2, 3].

In South and South-East Asia, including Taiwan, where betel quid (BQ) chewing is common, BQ contributes to a large proportion of HNC cases [4]. The International Agency for Research on Cancer has classified BQ without or with added tobacco and areca nut as carcinogenic to humans (group 1 agent) [5, 6].

Three forms of BQ are available in Taiwan: 1)”Betel quid”: two halves of an unripe areca nut and white slaked lime wrapped in betel leaf; 2)”Lao-hwa quid”: a piece of betel inflorescence and red lime inserted into an unripe areca nut; and 3) “Stem quid”: this is similar to the “lao-hwa quid” except that betel inflorescence is replaced with betel stem. “Stem quid” is rare and is consumed mostly by the aboriginal Taiwanese [5, 6]. Despite public health efforts, BQ use remains a serious problem in Taiwan.

Although BQ is an established risk factor for HNC, current literature has several insufficiencies: 1) most studies did not examine the different types of BQ; 2) many studies did not adjust for potential confounders, including alcohol and cigarette; 3) only a few studies examined the association between BQ and HNC by sites; 4) inadequate data on dose-response; and 5) lack of data on HNC risk after BQ cessation [7]. We conducted a comprehensive analysis to address these insufficiencies. In addition, a study by Lee et al. reported that BQ chewing was associated with an earlier onset of HNC, suggesting that BQ chewing may be associated with a shorter tumor promotion period [4]. Therefore, we also conducted analysis to compare the age of HNC diagnosis between HNC patients who were BQ chewers and HNC patients who did not chew BQ.

## Material and Methods

The institutional review boards of the National Cheng Kung University Hospital and the National Health Research Institutes approved this study. Every study participant signed an informed consent.

### Study subjects

Subjects were recruited from the Department of Otolaryngology and the Department of Stomatology at the National Cheng Kung University Hospital from September 1, 2010 to June 30, 2014. Cases were patients newly diagnosed with pathologically confirmed squamous cell carcinoma of head and neck (oral cavity, oropharynx, hypopharynx, and larynx), who had no previous diagnosis of cancer and were aged 20 or older. Frequency-matched [by age (±5 years) and sex] controls were recruited from the same departments. Controls were patients who underwent surgery for non-cancerous diseases unrelated to the use of alcohol, BQ, and cigarette and had no history of cancer diagnosis.

### Data collection

Each participant was interviewed about BQ chewing using a standardized questionnaire. Each participant was first asked whether he/she had chewed BQ at least once a day for six consecutive months in his/her lifetime. This is to define BQ chewers as those who chew BQ as a habit. Those with a positive response were further asked the followings: 1) starting age; 2) quitting age if the subject had quit BQ for > 6 months; 3) types of BQ; 4) number of BQ per day; and 5) average time (in minutes) for chewing each BQ. Data on sex, age, educational level, and use of alcohol and cigarette were also collected.

### Statistical analysis

Because only 1 woman among the 51 female participants (2%) was BQ users, women (28 HNC cases and 23 controls) were excluded from the current analysis. The distributions of age, education, and use of alcohol and cigarette between cases and controls were compared using t-tests (for continuous variables) and chi-squared tests (for categorical variables). Odds ratio (OR) and 95% confidence interval (CI) of HNC associated with BQ were estimated using multivariable unconditional logistic regression, adjusted for age, education, and consumption of alcohol and cigarette. The analyses were first performed combining all HNC sites and then by each site and subsites of oral cavity (tongue, buccal mucosa, gingiva, and others) and pharynx (tonsil + tongue base, other oropharynx, and hypopharynx).

The association between HNC and BQ was examined: 1) by BQ chewing status: never, former (stopped for > 6 months), or current; 2) by BQ types; 3) by pack-years with one pack-year of BQ chewing = 1 pack of BQs (20 quids) per day x 1 year; 4) by the total time of BQ chewing = number of BQs per day x average chewing time (in minutes) for each quid x days of chewing; and 5) by the total years of BQ cessation.

Two methods were used to analyze the continuous variables (BQ pack-years, total time of chewing, and total years of cessation). In the first method, these variables were analyzed as equal-parts categories (except for the highest category): BQ pack-years in 10-pack-year categories, total time of BQ chewing in 200,000-minute categories, and total years of BQ cessation in 10-year categories. In the second method, these variables were analyzed in their original forms. First, the linearity assumption was evaluated with the restricted cubic spline function using the %RCS_REG SAS macro [8]. Since no evidence of non-linearity was detected for the total years of BQ cessation, it was analyzed as a linear variable. Because non-linearity was detected for BQ pack-years and total time of BQ chewing, logistic B-spline regression models were performed using the SAS macros %regspline, %regspline_lot, and %regspline_subset [9]. Spline models were built with knots at consecutive tertiles (two knots), quartiles (three knots), and quintiles (four knots) of the continuous BQ variables with different degrees of freedom. The best models were selected with the smallest Akaike’s Information Criterion.

To assess the joint effect of BQ, alcohol and cigarette, the HNC risk was analyzed by the combinations of BQ, alcohol, or cigarette use. T-test was performed to compare the mean age of HNC diagnosis between BQ users and non-BQ users. Multiple linear regression was performed to assess the association between the age of HNC diagnosis and BQ use, adjusted for education, alcohol drinking, and cigarette smoking.

To quantify the contribution of BQ to HNC risk, population attributable risk percent (PAR%) was calculated using the formula: PAR% = [(P_e_(RR-1))/(P_e_(RR-1) + 1)] x 100 [10]. Relative risk (RR) was estimated using the OR of HNC associated with BQ chewing. The prevalence of BQ use (P_e_) was estimated by the percentage of ever BQ users among controls.

## Results

This analysis included 487 male HNC cases (313 oral cancers, 119 oro- and hypopharyngeal cancers, and 55 laryngeal cancers) and 617 male controls with a participation of 77% and 86% for the cases and controls, respectively. The distribution of the clinical diagnoses among controls is shown in S1 Table. Cases and controls were similar in age (Table 1). Controls had higher educational levels and consumed less alcohol and cigarette than cases.

**Table 1:** Demographic and lifestyle characteristics of the head and neck cancer patients and control subjects.

Characteristics	Cases N = 487 n (%)	Controls N = 617 n (%)	*P*
**Age (years)**			
Mean (SE)	54.7 (0.5)	54.1 (0.4)	0.33
**Education**			
≦ Elementary school	140 (28.8)	97 (15.7)	<0.0001
Junior high	154 (31.6)	123 (19.9)	
High school/Technical school	148 (30.4)	225 (36.5)	
Some college or more	45 (9.2)	172 (27.9)	
**Alcohol**			
Never + occasional	139 (28.5)	325 (52.7)	<0.0001
Former regular	67 (13.8)	76 (12.3)	
Current regular	281 (57.7)	216 (35.0)	
Never	122 (25.0)	277 (44.9)	<0.0001
1 drink or less per month	17 (3.5)	48 (7.8)	
1–2 drinks per week	19 (3.9)	43 (7.0)	
3–5 drinks per week	33 (6.8)	51 (8.3)	
Daily drinkers	283 (58.1)	192 (31.0)	
Unknown	13 (2.7)	6 (1.0)	
**Cigarette**			
Never	45 (9.2)	180 (29.2)	<0.0001
Former	93 (19.1)	133 (21.5)	
Current	348 (71.5)	303 (49.1)	
Unknown	1 (0.2)	1 (0.2)	
Never	45 (9.2)	180 (29.2)	<0.0001
0.1–9.9 pack-years	25 (5.1)	50 (8.1)	
10.0–19.9 pack-years	55 (11.3)	79 (12.8)	
20.0–29.9 pack-years	88 (18.1)	90 (14.6)	
30.0 or more pack-years	267 (54.8)	213 (34.5)	
Unknown	7 (1.5)	5 (0.8)	
Pack-years (SE)	36.8 (1.2)	24.4 (1.1)	<0.0001

Abbreviations: N = number; SE = standard error

BQ chewing was associated with an increased HNC risk (former chewer: OR = 4.20, 95% CI: 2.97–5.95; current chewer: OR = 6.09, 95% CI: 4.08–9.08) (Table 2). BQ chewing was associated most strongly in magnitude with oral cancer followed by pharyngeal cancer and not statistically significant for laryngeal cancer. The association between BQ and HNC was similar by BQ types.

**Table 2:** The association between betel quid use and risk of head and neck cancer overall and by sites.

Characteristics	Controls N = 617 n (%)	All head and neck cancer N = 487 n (%)	OR (95% CI)^a^	Oral cavity N = 313 n (%)	OR (95% CI)^a^	Pharynx N = 119 n (%)	OR (95% CI)^a^	Larynx N = 55 n (%)	OR (95% CI)^a^
**Betel quid chewing status**									
Never	446 (72.3)	127 (26.1)	Referent	67 (21.4)	Referent	31 (26.1)	Referent	29 (52.7)	Referent
Former	105 (17.0)	192 (39.4)	4.20 (2.97–5.95)	133 (42.5)	6.43 (4.25–9.73)	43 (36.1)	2.87 (1.61–5.13)	16 (29.1)	1.24 (0.57–2.71)
Current	66 (10.7)	168 (34.5)	6.09 (4.08–9.08)	113 (36.1)	8.05 (5.10–12.71)	45 (37.8)	4.80 (2.57–8.99)	10 (18.2)	2.38 (0.94–6.06)
Ever (former + current)	171 (27.7)	360 (73.9)	4.85 (3.54–6.64)	246 (78.6)	7.03 (4.81–10.28)	88 (73.9)	3.54 (2.09–5.98)	26 (47.3)	1.53 (0.76–3.07)
**Types of betel quid chewed**									
Never	446 (72.3)	127 (26.1)	Referent	67 (21.4)	Referent	31 (26.0)	Referent	29 (52.7)	Referent
With betel leaf (Betel quid)	70 (11.3)	147 (30.2)	4.73 (3.19–7.01)	96 (30.7)	6.31 (3.98–10.00)	36 (30.3)	3.39 (1.80–6.35)	15 (27.3)	2.74 (1.17–6.41)
With betel inflorescence (Lao-hwa)	21 (3.4)	39 (8.0)	4.42 (2.44–7.98)	28 (8.9)	6.72 (3.48–12.99)	9 (7.6)	3.65 (1.40–9.47)	2 (3.6)	0.34 (0.10–2.53)
Both types	79 (12.8)	172 (35.4)	5.10 (3.52–7.41)	122 (39.0)	7.81 (5.07–12.04)	41 (34.4)	3.47 (1.90–6.34)	9 (16.4)	1.24 (0.50–3.07)
Unknown	1 (0.2)	2 (0.3)	--	0 (0.0)	--	2 (1.7)	--	0 (0.0)	--
**Betel pack-years**^b^									
Never	446 (72.3)	127 (26.1)	Referent	67 (21.4)	Referent	31 (26.0)	Referent	29 (52.7)	Referent
0.1–9.9 pack-years	56 (9.1)	70 (14.4)	3.28 (2.14–5.04)	52 (16.6)	5.01 (3.07–8.18)	14 (11.8)	2.00 (0.94–4.22)	4 (7.3)	0.94 (0.29–3.02)
10.0–19.9 pack-years	31 (5.0)	59 (12.1)	4.69 (2.83–7.79)	45 (14.4)	7.19 (4.10–12.61)	11 (9.2)	2.33 (1.00–5.44)	3 (5.4)	1.00 (0.26–3.89)
20.0–29.9 pack-years	27 (4.4)	55 (11.3)	4.63 (2.68–7.99)	27 (8.6)	4.67 (2.43–8.96)	19 (16.0)	5.82 (2.61–12.98)	9 (16.4)	3.13 (1.09–9.00)
30.0 or more pack-years	55 (8.9)	158 (32.4)	6.41 (4.22–9.76)	106 (33.9)	9.51 (5.82–15.52)	43 (36.2)	5.51 (2.86–10.61)	9 (16.4)	1.54 (0.60–3.96)
Unknown	2 (0.3)	18 (3.7)	--	16 (5.1)	--	1 (0.8)	--	1 (1.8)	--
Ordinal^c^			1.58 (1.43–1.75)		1.67 (1.49–1.87)		1.55 (1.32–1.81)		1.18 (0.95–1.46)
Test for trend^c^			<0.0001		<0.0001		<0.0001		0.14
**Total time chewed (Thousand minutes)**									
0	446 (72.3)	127 (26.1)	Referent	67 (21.4)	Referent	31 (26.0)	Referent	29 (52.7)	Referent
<200	42 (6.8)	39 (8.0)	2.59 (1.57–4.26)	29 (9.3)	3.74 (2.14–6.53)	8 (6.7)	1.62 (0.67–3.92)	2 (3.6)	0.85 (0.19–3.88)
201–399	20 (3.2)	38 (7.8)	5.15 (2.83–9.37)	25 (8.0)	6.62 (3.42–12.82)	9 (7.6)	3.41 (1.35–8.66)	4 (7.3)	3.04 (0.88–10.56)
401–599	19 (3.1)	25 (5.1)	3.66 (1.90–7.05)	18 (5.7)	4.79 (2.34–9.83)	5 (4.2)	2.10 (0.69–6.36)	2 (3.6)	1.76 (0.35–8.86)
600 or more	83 (13.5)	220 (45.2)	6.43 (4.53–9.13)	143 (45.7)	8.20 (5.46–12.33)	61 (51.3)	5.56 (3.21–9.63)	16 (29.1)	3.20 (1.49–6.89)
Unknown	7 (1.1)	38 (7.8)	--	31 (9.9)	--	5 (4.2)	--	2 (3.6)	--
Ordinal^c^			1.58 (1.45–1.72)		1.65 (1.50–1.82)		1.51 (1.32–1.73)		1.35 (1.12–1.64)
Test for trend^c^			<0.0001		<0.0001		<0.0001		0.002
**Time since quitting**									
Current chewer	66 (10.7)	168 (34.5)	Referent	113 (36.1)	Referent	45 (37.8)	Referent	10 (18.2)	Referent
0.0–9.9 years	56 (9.1)	103 (21.2)	0.69 (0.44–1.07)	67 (21.4)	0.72 (0.44–1.17)	28 (23.5)	0.74 (0.39–1.42)	8 (14.6)	0.62 (0.22–1.81)
10.0–19.9 years	23 (3.7)	63 (12.9)	1.10 (0.62–1.97)	48 (15.3)	1.42 (0.77–2.61)	9 (7.6)	0.63 (0.24–1.61)	6 (10.9)	0.73 (0.19–2.71)
20.0 years or more	25 (4.0)	23 (4.7)	0.27 (0.13–0.53)	15 (4.8)	0.34 (0.16–0.73)	6 (5.0)	0.26 (0.09–0.78)	2 (3.6)	0.20 (0.04–1.08)
Never chewer	446 (72.3)	127 (26.1)	0.16 (0.11–0.24)	67 (21.4)	0.12 (0.08–0.20)	31 (26.1)	0.20 (0.11–0.38)	29 (52.7)	0.41 (0.16–1.05)
Unknown	1 (0.2)	3 (0.6)	--	3 (1.0)	--	0 (0.0)	--	0 (0.0)	--
Ordinal^c^^,^^d^			0.83 (0.67–1.02)		0.87 (0.70–1.09)		0.72 (0.52–0.99)		0.74 (0.44–1.24)
Test for trend^c^^,^^d^			0.08		0.23		0.04		0.25
Every 1 year^d^			0.972 (0.950–0.995)		0.976 (0.952–1.001)		0.967 (0.933–1.001)		0.955 (0.901–1.011)

Abbreviations: N = number; CI = confidence interval; OR = odds ratio

^a^ OR and 95% CI were calculated using unconditional logistic regression, adjusted for age, education, cigarette smoking (pack-year categories), and alcohol drinking (frequency)

^b^ 1 pack year = 20 betel quids/per day x 1 year

^c^ Those with an unknown value were excluded from the analysis.

^d^ Never betel quid chewers were excluded from the analysis

**Fig 1:**
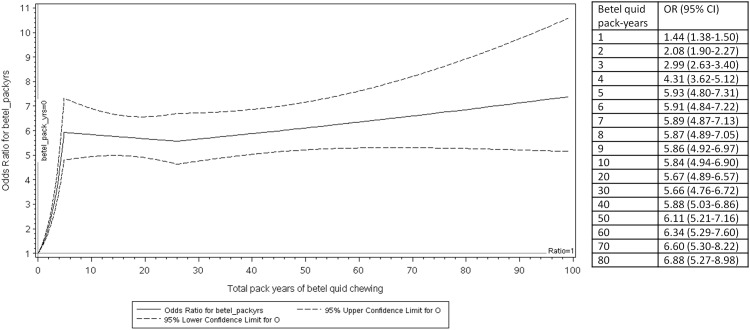
The association between pack-year of betel quid chewing and risk of head and neck cancer. This figure shows the non-linear relationship between pack-year of betel quid chewing and risk of head and neck cancer. The middle solid line represents the odds ratios, the upper dashed line represents the 95% upper confidence intervals, and the lower dashed line represents the 95% lower confidence intervals.

**Fig 2:**
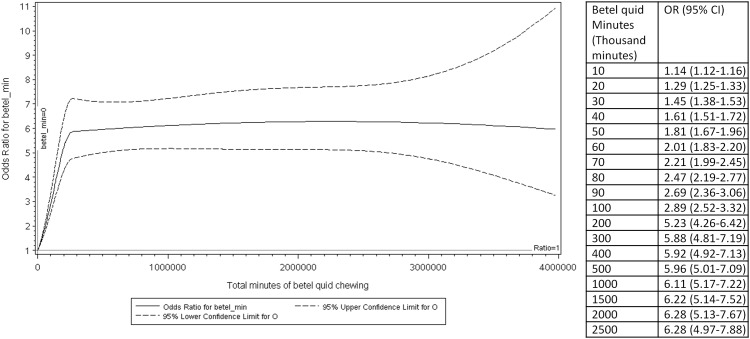
The association between total minute of betel quid chewing and risk of head and neck cancer. This figure shows the non-linear relationship between total minute of betel quid chewing and risk of head and neck cancer. The middle solid line represents the odds ratios, the upper dashed line represents the 95% upper confidence intervals, and the lower dashed line represents the 95% lower confidence intervals.

When analyzed in 10-pack-year categories, the association between BQ and HNC risk showed a significantly positive trend (Table 2). In the logistic B-spline regression model (Fig 1), the HNC risk increased sharply for the first 5 pack-years and the increasing trend attenuated thereafter. When analyzed in 200,000-minute categories for the total time of BQ chewing, there was a positive trend in the association between the total time of BQ chewing and HNC risk (Table 2). In the logistic B-spline regression model (Fig 2), the HNC risk increased sharply up to 200,000 minutes and plateaued off thereafter.

When analyzed in 10-year categories, a significantly reduced HNC risk was observed only for BQ cessation ≧20 years (OR = 0.27, 95% CI: 0.13–0.53), but the risk was still greater than non-BQ chewers (Table 2). As a continuous variable, each year of BQ cessation was associated with a 2.9% reduction in HNC risk (OR = 0.972, 95% CI: 0.950–0.995). Every year of BQ cessation was associated with a 4.0% reduction in HNC risk (OR = 0.962, 95% CI: 0.931–0.993) for those who chewed ≦19 pack-years (the median BQ pack-year for the BQ chewers among controls), while no reduction of HNC risk was observed for those who chewed >19 pack-years (OR = 1.015, 95% CI: 0.959–1.073). Similarly, every year of BQ cessation was associated with a 3.3% reduction in HNC risk (OR = 0.968, 95% CI: 0.934–1.003) for those who chewed ≦620,594 minutes (the median total time of BQ chewing for the BQ chewers among controls), while no reduction of HNC risk was observed for those who chewed >620,594 minutes (OR = 1.011, 95% CI: 0.959–1.065).

By eliminating BQ chewing in Taiwan, we estimated that 51.6% of HNCs, 62.6% of oral cancers, and 41.3% of oropharyngeal + hypopharyngeal cancers may be prevented. PAR% of BQ was not calculated for laryngeal cancer because of the non-significant association.

For subsite analysis, the highest risk associated with BQ occurred in cancers of the buccal mucosa (OR = 12.08, 95% CI: 6.26–23.31) and gingiva (OR = 12.68, 95% CI: 3.35–47.99) (Table 3). Compared to buccal cancer and gingival cancer, the increased risk in cancers of tonsil + tongue base (OR = 3.11, 95% CI: 1.25–7.76) or hypopharynx (OR = 3.28, 95% CI: 1.62–6.64) was smaller in magnitude.

**Table 3:** The association between betel quid use (ever vs. never) and risk of oral or pharyngeal cancer by subsites.

	OR (95% CI)^a^
**Oral Cavity (N = 313)**	
Tongue (n = 121)	4.23 (2.53–7.09)
Buccal mucosa (n = 114)	12.08 (6.26–23.31)
Gingiva (n = 26)	12.68 (3.35–47.99)
Hard palate (n = 11) + floor of the mouth (n = 13) + lip (N = 10) and unspecified subsites of oral cavity (N = 18)	9.65 (4.14–22.52)
**Pharynx (N = 119)**	
Oropharynx: tonsil + tongue base (n = 31)	3.11 (1.25–7.76)
Other oropharynx (n = 29)^b^	7.16 (2.09–24.54)
Hypopharynx (n = 59)	3.28 (1.62–6.64)

Abbreviations: CI = confidence interval; OR = odds ratio

^a^ OR and 95% CI were calculated using unconditional logistic regression, adjusted for age, education, cigarette smoking (pack-year categories), and alcohol drinking (frequency)

^b^ Other oropharynx = soft palate (n = 2) and unspecified subsites of oropharynx (n = 27)

We observed that the risk of HNC was higher among individuals who consumed cigarette and alcohol in addition to BQ (Table 4). Compared to no use of alcohol, BQ, and cigarette, use of BQ plus either alcohol or cigarette had an OR = 8.12 (95% CI: 4.49–14.70), while use of alcohol, BQ, and cigarette had an OR = 14.35 (95% CI: 8.41–24.49).

**Table 4:** The association between the use of alcohol, betel quid, and cigarette and risk of head and neck cancer.

Use of alcohol, betel quid, and cigarette	Cases n (%)	Controls n (%)	OR (95% CI)^a^
No use	20 (4.1)	141 (22.9)	Referent
Betel quid plus either alcohol or cigarette	87 (17.9)	57 (9.2)	8.12 (4.49–14.70)
Betel quid plus both alcohol and cigarette	270 (55.6)	112 (18.2)	14.35 (8.41–24.49)
Other combinations^b^	109 (22.4)	306 (49.7)	2.30 (1.37–3.89)

Abbreviations: CI = confidence interval; OR = odds ratio

^a^ OR and 95% CI were calculated using unconditional logistic regression, adjusted for age and education.

^b^ Other combinations included other combinations of alcohol, betel quid, and cigarette, including alcohol only, betel quid only, cigarette only, and alcohol + cigarette.

Finally, we analyzed the association between BQ chewing and age of HNC diagnosis. BQ users were significantly younger in the mean age of HNC diagnosis than non-BQ users (mean age: 53.35 years vs. 58.62 years, *P* < 0.0001). After adjusting for education, alcohol, and cigarette, BQ users still had a younger mean age of diagnosis than non-BQ users by 5.78 years (*P* < 0.0001). HNC patients who were regular alcohol drinkers (at least once a week) were on average 2.22 years younger in age of HNC diagnosis compared to those who were occasional/non-drinkers (*P* = 0.02). Smoking was not associated with the age of HNC diagnosis (*P* = 0.98). Patients with oral cancer (mean age = 53.59 years) and pharyngeal cancer (mean age = 53.90 years) were significantly younger than laryngeal cancer patients (mean age = 62.99 years) (*P* < 0.0001). HNC patients who used BQ, alcohol, and cigarette had a significantly younger age of HNC diagnosis than HNC patients with other patterns of alcohol, BQ, and cigarette consumption (52.72 years vs. 57.22 years, *P* < 0.0001).

## Discussion

Our analysis showed a non-linear positive association between BQ and HNC. The highest HNC risk associated with BQ occurred in buccal mucosa and gingiva. Every year of BQ cessation was associated with a 2.9% HNC risk reduction, but the risk remained higher than that of non-BQ chewers even after 20 years of BQ cessation. BQ cessation was associated with a reduced HNC risk more for those who chewed less in amount and duration. Eliminating BQ chewing may prevent 51.6% of HNCs in Taiwan. The highest risk of HNC was observed who used BQ, alcohol, and cigarette.

Consistent with literature, we observed a positive association between BQ and HNC. Our spline models showed that the HNC risk increased sharply at the lower BQ exposure and attenuated at higher BQ exposure. This may reflect the saturation of the key enzymes involved in the carcinogenesis [11]. Alternatively, it may be caused by the misclassification of BQ exposure, particularly at the higher exposure levels [11]. The only other Taiwanese study that examined the non-linear relationship between BQ and HNC also reported dose-response curves similar to ours, with risk of oral cancer and pharyngeal cancer increasing sharply from 0 to 15 BQs per day and attenuated after 15 BQs per day [4]. In a meta-analysis by Guha *et al*., a dose-response curve similar to ours was observed for studies from India for number of BQ per day and years of BQ chewing, whereas the meta-analysis of studies from Taiwan showed a linear positive association between number of BQ per day and HNC [7]. A limitation of the meta-analysis is that the spline models were built using the reported relative risks based on categorized BQ exposures and not the original data points. Another strength of our dose-response analysis is that we used BQ pack-years and total time of BQ consumption to capture both the amount and the duration of BQ chewing. More studies with original data and optimal treatment of continuous variables are needed to evaluate the dose-response relationship between BQ and HNC.

Our study showed no difference in the association between BQs and HNC by BQ types. Lee *et al*. showed that BQ with betel inflorescence was associated with a higher HNC risk than BQ without betel inflorescence [4]. Lee *et al*. suggested that the higher HNC risk could be attributed to safrole, a possible human carcinogen (Class 2B), in the betel inflorescence [5]. Despite such plausible explanation, our study did not observe a higher HNC risk for BQ with betel inflorescence. Regardless of the inconsistent results, the public health message is clear that all types of BQ increase HNC risk, although the difference in the magnitude needs further investigation.

Our results showed that the risk associated with BQ was the highest for oral cancer followed by pharyngeal cancer, indicating that the risk is the highest at the sites of direct BQ contact. Further support came from the subsite analysis of oral cavity with the highest cancer risk occurring in buccal mucosa, where BQ is often placed, and in gingiva, which is involved in chewing BQ. A meta-analysis by Guha et al. also showed that BQ, particularly with added tobacco, was more strongly associated with cancer of the buccal mucosa [7]. Among the subsites of the pharynx, the weakest association with BQ was for cancers of the tonsil and tongue base, which are often associated with human papillomavirus [12].

We found that HNC patients who used BQ were significantly younger than HNC patients who were non-BQ users. Furthermore, patients with oral cancer and pharyngeal cancer were significantly younger than laryngeal cancer patients. Since BQ was more strongly associated with oral and pharyngeal cancers than with laryngeal cancer, our results further supported the association between BQ use and the younger age of HNC diagnosis. This pattern of age distribution by HNC sites has been reported by other Taiwanese studies [4, 13]. This suggests that BQ may induce a shorter tumor promotion, resulting in an earlier HNC development. One may wonder if the younger age of HNC diagnosis associated with BQ chewing was because of the younger starting age of BQ chewing. In our study, the average starting age for BQ chewing was 23.2 years old, whereas the average starting age for cigarette smoking was 20.2 years old. In addition, among the subjects that smoked cigarette and chewed BQ, 44% started smoking cigarette before starting chewing BQ, 51% started both at the same age, and only 5% started chewing BQ before starting smoking cigarette. Therefore, the shorter tumor promotion time of BQ according to our analysis was not due to the younger starting age of BQ chewing.

In our study, every year of BQ cessation was associated with a 2.9% HNC risk reduction; however, even after 20 years of BQ cessation, the risk was still higher than that of non-BQ chewers. Only two previous studies, both from India, evaluated the effect of BQ cessation on HNC risk [14, 15]. Balaram *et al*. did not observe a significant oral cancer risk reduction even after ≥10 years of BQ cessation; however, numbers of subjects with ≥10 years of BQ cessation were small (Men: 14 cases and 6 controls; women 17 cases and 3 controls) [14]. Znaor *et al*. showed that BQ cessation was associated with a non-statistically significant HNC risk reduction only after ≥10 years of BQ cessation [15]. Tobacco is frequently added to BQ in India and never in Taiwan. It is possible that the addition of tobacco makes BQ more carcinogenic and studies with a larger sample size and more subjects with a longer BQ cessation are needed to have a sufficient statistical power to detect a significant effect of BQ cessation. Although more studies are needed, the results of our study and the two previous studies suggest that it may take a long period of BQ cessation (> 20 years) to reverse the damage incurred by BQ chewing. Even so, our study indicated that the benefit of BQ cessation is cumulative (2.9% reduction in HNC risk per year of BQ cessation) and thus BQ cessation should be highly promoted.

We found that BQ cessation appeared more effective in reducing HNC risk for those that chewed less in amount and duration. This indicated that at certain point, the damage caused by BQ may be too severe or irreversible for BQ cessation to overcome. For example, BQ is a major risk factor for oral submucous fibrosis, an irreversible potentially malignant disorder that is characterized by juxta-epithelial inflammation and progressive fibrosis of the submucosal tissues [16]. The malignant transformation rate of OSF was estimated at 7.6% for a follow-up period of 17 years [17]. To our knowledge, our study is the first to show that the amount of BQ consumed may influence the effect of BQ cessation on HNC risk. More studies are needed to confirm our finding. In addition, our study was not able to indicate the amount or the duration of BQ consumption that may possibly lead to the irreversible process for developing HNC and more investigations are needed to clarify this point. In the mean time, our results suggest that it would be important to encourage BQ cessation as early as possible to avoid the development of irreversible damage.

Consistent with previous studies [4, 15, 18–20], we observed that the highest risk of HNC was among individuals who consumed BQ, cigarette, and alcohol. Alcohol may act as solvent for environmental carcinogens, including those in BQ and cigarette, to facilitate the entry of these carcinogens into the mucosal cells of head and neck [21]. Carcinogens from BQ, cigarette, and alcohol may act together to generate higher genotoxicity. Bharali *et al*. found that alcohol and BQ enhanced the formation of cigarette-induced micronuclei (a marker for genotoxicity) in the buccal epithelia [22].

This study has several limitations. In a hospital-based case-control study, it is difficult to determine whether the cases and controls are from the same source population. Several measures were taken to minimize selection bias. First, our recruiting hospital is a major medical center in Tainan city, where >90% of our study subjects were from. We performed sensitivity analysis excluding subjects living outside of Tainan city, and the results were similar (data not shown). Second, we excluded from controls those who were admitted with diseases related to the consumption of alcohol, BQ, or cigarette. Among our controls, the prevalence of current BQ chewer was 10.7%. According to a population-based survey in 2009, the percentage of current BQ chewer among men aged ≧20 years living in Tainan city was 12.3% [3]. The slightly lower percentage of current BQ chewer in our controls compared to that of the population survey could be due to random variation or due to the declining BQ use during our recruitment period; therefore, we consider our controls to be representative of the source population in BQ consumption. Recall bias can be an issue in a case-control study. HNC patients could have ruminated more about their BQ chewing experience and over-reported their BQ consumption, resulting in overestimation of the positive association between BQ and HNC. However, in a cohort study of Taiwanese men, Hsu *et al*. reported that BQ was associated with an increased risk of oral cancer [Hazard ratio (HR) = 5.77, 95% CI: 3.60–9.25] and pharyngeal cancer (HR = 3.30, 95% CI: 1.90–5.74) [19]. Cohort studies are less affected by recall bias, because the exposure information is obtained before the disease occurrence. The relative risk estimates of our case-control study [Oral cancer OR = 7.03, 95% CI: 4.81–10.28] and pharyngeal cancer (HR = 3.54, 95% CI: 2.09–5.98) were similar to those estimated by Hsu *et al*., [19] suggesting that our study was minimally affected by recall bias.

The major strength of our study is that it fills the insufficiencies in the literature. First, few studies examined the dose-response relationship between BQ and HNC. Those that did mostly assumed a linear relationship between BQ and HNC risk and categorized continuous data. A linear model may not always represent the biology of an exposure-response relationship. Categorization of a continuous variable can suffer from information loss and misclassification. To avoid these pitfalls, we used spline models to fit our data and we believe that our models better represent the dose-response relationship between BQ and HNC. In addition, we used BQ pack-years and total time of BQ consumption to capture the amount and the duration of BQ consumption. Second, our study is only the second study with a large enough sample size to examine association between HNC risk and the different types of BQ in Taiwan. Third, we were able to evaluate the association between BQ and HNC by sites and subsites. Finally, our study is only the third one to evaluate the effect of BQ cessation on HNC risk and the first with a sufficient statistical power to show a statistically significant HNC risk reduction associated with BQ cessation.

Overall, we found a positive association between BQ and HNC regardless of the BQ types. Every year of BQ cessation was associated with a 2.9% HNC risk reduction; however, the risk remained higher than non-BQ chewers even after 20 years of BQ cessation, indicating the importance to prevent the initiation of BQ chewing. BQ cessation appeared more effective in reducing HNC risk for those who chewed less in amount and duration. BQ cessation should be encouraged, particularly for those who are at the early years of BQ consumption to maximize the benefit of BQ cessation in reducing HNC risk.

## Supporting Information

S1 Table The distribution of clinical diagnoses among controls. (DOC)
