# Supplementary material for: A Comprehensive Analysis on the Association between Tobacco-Free Betel Quid and Risk of Head and Neck Cancer in Taiwanese Men
Source: PLoS One. 2016 Oct 25;11(10):e0164937. doi: 10.1371/journal.pone.0164937 (PMC5079549; doi:10.1371/journal.pone.0164937)
Supplement: S1 Table — (DOC) [file pone.0164937.s001.doc]

Supplementary Table 1. The distribution of clinical diagnoses among controls

| **Clinical diagnosis** | **N = 617**  **n** |
| --- | --- |
| [Antrochoanal poly](http://radiopaedia.org/articles/antrochoanal-polyp-1)p | 3 |
| Benign ethmoid tumor | 1 |
| Benign hypopharyngeal lesions | 6 |
| Benign laryngeal lesions | 40 |
| Benign maxillary sinus lesions | 4 |
| Benign nasopharyngeal tumor | 4 |
| Benign oral lesions | 30 |
| Benign oropharyngeal lesions | 15 |
| Benign salivary gland tumor | 86 |
| Benign vocal cord lesions | 84 |
| Chest abscess | 1 |
| Cholesteatoma | 6 |
| Chronic otitis media | 29 |
| Chronic rhinitis | 17 |
| Chronic sinusitis | 107 |
| Deep neck infection | 4 |
| Epiglottic cyst | 12 |
| Epistaxis | 1 |
| Esophageal stenosis | 1 |
| Ethmoid mucocele | 2 |
| Incomplete glottis closure | 2 |
| Laryngocele | 1 |
| Middle turbinate headache syndrome | 2 |
| Nasal polyp | 1 |
| Nasal septum deviation | 1 |
| Nasal synechiae | 1 |
| Neck lipoma | 35 |
| Neck lymphangioma | 1 |
| Obstructive sleep apnea | 10 |
| Osteoma | 1 |
| Preauricular sinus | 1 |
| Sialolithiasis | 15 |
| Thyroglossal duct cyst | 10 |
| Tonsillitis | 7 |
| Tracheal granuloma | 1 |
| Vocal cord palsy | 6 |
| Vocal cord polyp | 68 |
| Wegener’s granulomatosis | 1 |
